# Hydrophilic Dual Layer Hollow Fiber Membranes for Ultrafiltration

**DOI:** 10.3390/membranes10070143

**Published:** 2020-07-06

**Authors:** Lara Grünig, Ulrich A. Handge, Joachim Koll, Oliver Gronwald, Martin Weber, Birgit Hankiewicz, Nico Scharnagl, Volker Abetz

**Affiliations:** 1 Institute of Polymer Research, Helmholtz-Zentrum Geesthacht, Max-Planck-Strasse 1, 21502 Geesthacht, Germany; lara.gruenig@hzg.de (L.G.); joachim.koll@hzg.de (J.K.); volker.abetz@hzg.de (V.A.); 2 Advanced Materials & Systems Research, Performance Polymer Blends & Membranes, BASF SE, RAP/ES, 67056 Ludwigshafen, Germany; oliver.gronwald@basf.com (O.G.); martin.weber@basf.com (M.W.); 3 Institute of Physical Chemistry, Universität Hamburg, Martin-Luther-King-Platz 6, 20146 Hamburg, Germany; birgit.hankiewicz@chemie.uni-hamburg.de; 4 Institute of Materials Research, Magnesium Innovation Center (MagIC), Helmholtz-Zentrum Geesthacht, Max-Planck-Strasse 1, 21502 Geesthacht, Germany; nico.scharnagl@hzg.de

**Keywords:** poly(ether sulfone), hollow fiber membranes, triblock copolymers, hydrophilicity, fouling

## Abstract

In this study, a triblock copolymer was used as additive to fabricate new dual layer hollow fiber membranes with a hydrophilic active inner surface in order to improve their fouling resistance. The polymeric components of the solutions for membrane fabrication were poly(ether sulfone), poly(*N*-vinyl pyrrolidone), and the triblock copolymer. The additive consists of three blocks: a middle hydrophobic poly(ether sulfone) block and two outer hydrophilic alkyl poly(ethylene glycol) blocks. By varying the additive concentration in the solutions, it was possible to fabricate dual layer hollow fiber membranes that are characterized by a hydrophilic inner layer, a pure water permeance of over 1800 L/(m^2^ bar h) and a molecular weight cut-off of 100 kDa similar to commercial membranes. Contact angle and composition determination by XPS measurements revealed the hydrophilic character of the membranes, which improved with increasing additive concentration. Rheological, dynamic light scattering, transmission, and cloud point experiments elucidated the molecular interaction, precipitation, and spinning behavior of the solutions. The low-molecular weight additive reduces the solution viscosity and thus the average relaxation time. On the contrary, slow processes appear with increasing additive concentration in the scattering data. Furthermore, phase separation occurred at a lower non-solvent concentration and the precipitation time increased with increasing additive content. These effects revealed a coupling mechanism of the triblock copolymer with poly(*N*-vinyl pyrrolidone) in solution. The chosen process parameters as well as the additive solutions provide an easy and inexpensive way to create an antifouling protection layer in situ with established recipes of poly(ether sulfone) hollow fiber membranes. Therefore, the membranes are promising candidates for fast integration in the membrane industry.

## 1. Introduction

Ultrafiltration membranes, based on poly(ether sulfone) (PESU), are commonly used and well established in the water filtration industry. In non-solvent induced phase separation processes (NIPS) PESU forms membranes with uniformly distributed pores and a high porosity, which is sufficient in membrane technology to achieve not only reliable retention values, but also a high permeance. Especially in combination with other polymers for interconnection of the pores like poly(*N*-vinyl pyrrolidone) (PVP) or poly(ethylene glycol) (PEG) of high molecular weight, the pore diameter and the permeance of the membrane can be adjusted in a way that PESU is applicable in a wide range from gas separation to liquid filtration [1,2]. PESU is relatively resistant to chemicals and has a good thermal stability. However, the last aspect is not normally required for wastewater treatment.

Despite the mentioned benefits of PESU, two drawbacks have currently motivated research groups to make the membranes even more efficient. The first drawback is the relatively high hydrophobicity of poly(ether sulfone). Hydrophobicity on ultrafiltration membrane surfaces often results in membranes that are easily blocked by foulants and need to be chemically cleaned more often than hydrophilic membranes. The chemical cleaning leads to the second drawback of PESU, which is its sensitivity to halogens [3]. Since the chemical cleaning is usually done by sodium hypochlorite, the membrane not only is cleaned from foulants by this treatment, but also degradation of PESU occurs [3,4]. Consequently, the lifetime of the membrane is limited to a certain number of chemical cleaning cycles. If the necessity of chemical cleaning can be reduced due to better fouling resistance, the membrane’s lifetime can profit as well. Knowing these challenges, one can go to chemically more stable polymers like poly(vinylidene fluoride), poly(phenylene sulfone) or poly(tetrafluoro ethylene), which has been conducted by various groups [5,6,7,8]. However, these materials suffer from other drawbacks like low permeance and difficult processability, or even higher hydrophobicity and lower surface energy. Another possibility is to enhance the resistance to fouling processes on the actual well-established membrane. The second option is what this study aims to accomplish. Numerous efforts have been made during recent years to functionalize the active surface of PESU membranes and improve the fouling behavior of the membrane. The implementation of polycations, enzymes, photoactive agents or nanoparticles (NP) was the driving force of many studies and showed promising results [9,10,11,12,13,14]. Most recent research articles have been devoted to biomimetic, mussel-inspired surface engineering on polymeric membranes [15]. Various studies showed a decreasing effect of the fouling tendency when the active surface became more hydrophilic [16,17].

For industry purposes, one must keep in mind that hydrophilizing the surface of the membrane is only one aspect that characterizes an applicable anti-fouling additive. In water treatment processes, the additive must be nontoxic, strongly integrated into the membrane surface, and be of low-cost in order to be attractive for industry. In the case of integrated nanoparticles (NP), there are very few long-term studies on the effect of NP in human bodies, since those NP can dissolve potentially from the membrane structure into the drinking water. Even though there are promising reports on, for example, TiO_2_ or Ag nanoparticles, which can act antibacterially or photocatalytically, the risk of diseases caused by detached NP exists [18].

A recent publication proved the successful integration of amphiphilic block copolymers including hydrophilic building blocks into poly(phenylene sulfone) membranes [19] and their hydrophilic improvement along with less fouling tendency. In a previous project various additives with different hydrophilic units and molecular weights of the hydrophobic endgroups were investigated to a large extent [20]. Furthermore, additives based on an emulsifier with the commercial name Pluronic^®^ led to phase separated dope solutions at concentrations equal or higher than 3 wt% [21].

For these reasons a new kind of amphiphilic triblock copolymer was selected in this study. This polymer consists of a hydrophobic PESU block which is embedded by two hydrophilic blocks made of a monofunctional poly(alkylene oxide) [22]. This poly(alkylene glycol ether) (saturated C_16_-C_18_ alcohol with 80 ethylene oxide groups) is a non-ionic surfactant. The listed aspects lead to an additive that fulfills most of the required aspects for industrial processes: With the PESU-backbone the additives are tightly integrated into the membrane surface. In addition, the monofunctional poly(alkylene oxide) is hydrophilic and of very low toxicity. With this approach two positive effects should be accomplished at once: A membrane that is easier to clean by pure water backwash and a hydrophilic active membrane surface through the integration of poly(ethylene oxide) (PEO) units, of which the foulants in aqueous media are repelled instead of attracted. As mentioned before, an intensive study indicated already the efficiency of these kinds of additives by integrating a low additive amount (1.6 wt%) to the actual dope solution [20,21]. However, such a low additive content can only produce a limited amount of fouling resistance. Providing a membrane with a highly enriched additive layer is the driving force of this study with the aim to improve the already promising results.

## 2. Materials and Methods

### 2.1. Materials

**Figure 1:**
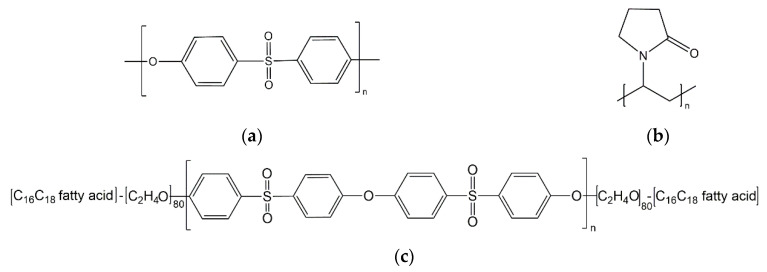
Chemical structure of (**a**) Poly(ether sulfone) (PESU); (**b**) Poly(*N*-vinyl pyrrolidone) (PVP); (**c**) Triblock copolymer PEO-PESU-PEO.

A commercial poly(ether sulfone) Ultrason^®^ E 3010 (weight average of the molecular weight *M_n_* approx. 58 kDa [21]) and poly(*N*-vinyl pyrrolidone) (PVP, tradename Luvitec^®^ K90, *M_n_* approx. 2000 kDa; both BASF SE, Ludwigshafen, Germany), were selected as polymer materials for membrane fabrication. An amphiphilic triblock copolymer, consisting of an inner PESU-block (*M_n_* approx. 5 kDa) and two outer poly(alkylene oxide)-blocks (*M_n_* of each outer block approx. 3.75 kDa, determination of averaged molecular weights see Ref [21,23]) were synthesized as described in the next section. The chemical structure of PESU, PVP, and the triblock copolymer poly(alkylene oxide)-PESU-poly(alkylene oxide) is presented in Figure 1.

Glycerol (purity > 99.5%) (Sigma-Aldrich, München, Germany) and *N*-methyl-2-pyrrolidone (NMP) (Merck, Darmstadt, Germany) worked as non-solvent and solvent, respectively. The post-treatment was done with sodium hypochlorite (12% Cl., aq.) and sodium bisulfite (>97%), both purchased from Roth GmbH & Co. KG (Karlsruhe, Germany). The amount of free chlorine in the aqueous sodium hypochlorite solution was determined via iodometry before use. For better exposure of the segregated additives, the membranes were washed with hot demineralized water in an annealing step, after post-treatment.

In this study, all percentages refer to wt%, if not specified otherwise. Outer dope solutions for the spinning process of ultrafiltration hollow fiber membranes as well as the additive solutions were prepared according to the compositions, which are listed in Table 1. Demineralized water was used as coagulation bath for the hollow fiber membranes.

### 2.2. Additive Synthesis

**Figure 2:**
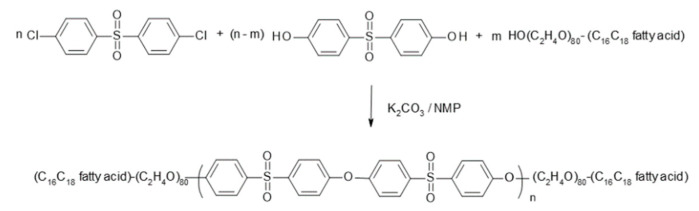
Synthesis mechanism of the amphiphilic triblock copolymer of type PEO-PESU-PEO.

The synthesis scheme is depicted in Figure 2. In a 4 L glass reactor fitted with a thermometer, a gas inlet tube and a Dean-Stark trap, 287.16 g (1.00 mol) 4,4′-dichloro-diphenylsulfone (DCDPS), 226.65 g (0.90 mol) 4,4’-dihydroxy-diphenylsulfone (DHDPS), 467.55 g (0.15 mol) α-hydroxy, ω- stearyl-poly(ethylene oxide), and 145.12 g (1.05 mol) potassium carbonate with a volume average particle size of 32.60 µm were suspended in 530 mL NMP under a nitrogen atmosphere. The mixture was heated to 190 °C within one hour and stirred at this temperature for nine hours. The water that was formed during the reaction was continuously removed by distillation. Subsequently, 970 mL NMP was added to the reactor to stop the reaction. The suspension was cooled to 80 °C and transferred into a pressure filter to separate the potassium chloride formed in the reaction by filtration.

In the next step, a part of the obtained polymer solution was precipitated in water, the resulting polymer beads were separated and extracted with hot water (85 °C) for 20 h. Afterwards, the beads were dried at 80 °C for 24 h at reduced pressure (<100 mbar). Finally, the filtered solution was stored in the dark. Analysis of ^1^H-NMR data of the polymer beads was conducted to determine the poly(ethylene oxide) share derived from the poly(ethylene oxide) building block. In addition, ^1^H- NMR was used to determine the number average molecular weight of the PESU block.

### 2.3. Preparation of Dope and Additive Solutions

For the outer shell of the hollow fiber membranes, a so-called “standard dope solution” was prepared (abbreviated as “PESU dope”). The inner shell was formed out of an “additive solution”. The corresponding recipes are listed in Table 1. All solutions were homogenized via a SpeedMixer™ DAC 800.2 (Hauschild & Co. KG, Hamm, Germany) at speeds of 500, 1000, and 1750 rpm within 30 min of mixing.

### 2.4. Viscoelastic, Diffusion and Precipitation Behaviour of the Solutions

In order to characterize the viscoelastic behavior of the solutions, the dynamic moduli *G*’ and *G*″ were determined via frequency sweeps. Starting with 100 rad/s, the angular frequency ω was varied from 0.01 to 100 rad/s, with a shear amplitude γ_0_ of 5%, which was within the linear viscoelastic range. All rheological measurements were performed using a rotational rheometer MCR 502 (Anton Paar, Graz, Austria). For the rheological experiments in shear, a Searle geometry was used. A volume of 14 mL of the polymer solution was transferred into the measurement cylinder for the investigations. The measurements were performed at a temperature of 60 °C, according to the spinning temperature.

In order to study diffusion phenomena, dynamic light scattering (DLS) experiments were performed with an ALV/CSG-3 Compact Goniometer-System (ALV-Laser Vertriebsgesellschaft GmbH, Langen, Germany) with an ALV/LSE-5003 Multiple Tau Digital Correlator. As light source a HeNe laser with a wavelength λ of 632.8 nm was used. Each sample was filled into a glass vial and tempered in the sample cell with a toluene bath at 60 °C for at least half an hour prior to the measurement. The toluene bath was tempered by a Julabo F25 thermostat working with a mixture of water and ethylene glycol. The accuracy of the temperature was 0.01 °C.

The scattering angle θ was varied between 40° to 140° in increments of 5° and each angle was measured twice for 120 s. The magnitude q of the scattering vector is related to the wavelength λ and the solvent refractive index n by

(1) $$q=\frac{4\pi n}{\lambda }\sin\left(\theta /2\right).$$

As refractive index of the solutions the refractive index of NMP (*n* = 1.4569) at 60 °C was used. The refractive index of glycerol is 1.4745, accordingly the difference in the solution mixture of NMP and glycerol is negligible in the further calculations. By applying a CONTIN algorithm to the field autocorrelation function [24], which was implemented in Matlab^®^, the relaxation time spectrum was determined.

**Figure 3:**
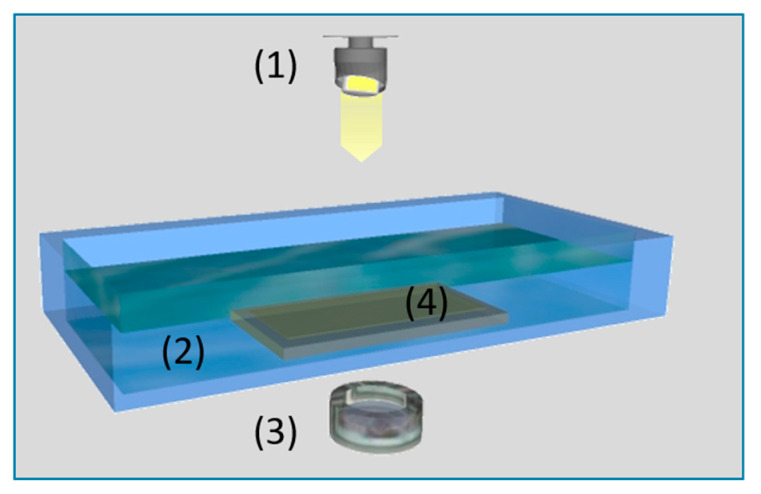
Setup for transmission experiments on precipitating polymer solutions.

Cloud point determinations were carried out at 25 and 60 °C to study the phase separation behavior. The solutions were stirred continuously while droplets of 15 µL of water were added. The next drop of water was added only if the solutions appeared transparent after dissolving the precipitated polymer in the solution. Every cloud point was determined by the average of at least two samples from each solution. Cloud point determinations can only reveal a trend on how much water is needed to precipitate the polymers in solution. This value is obviously not adjustable during the spinning process, since no such controlled amount of non-solvent can be added to the fibers. For this reason, the authors investigated specifically the precipitation time of each solution via transmission experiments. The experimental setup is depicted in Figure 3. A light source (1) is placed over a coagulation bath (2) containing water, and a photodetector (3) below the bath measures the light intensity that transmits through the precipitating membrane (4). The respective solution is hand-casted on a glass plate with a slit of 50 µm and a casting speed of 10 mm/s. Afterwards the glass plate is transferred into the water bath. Due to the precipitation of the polymers the film becomes non-transparent and consequently the detectable light intensity decreases.

### 2.5. Preparation of Hollow Fiber Membranes

**Figure 4:**
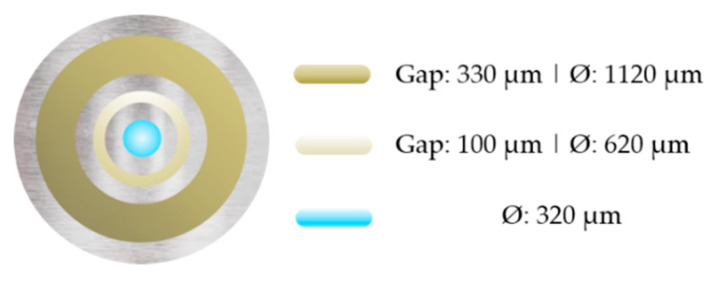
Scheme of the dual-layer spinneret. Inner hole: bore fluid, middle orifice: additive solution, outer orifice: dope solution. The given diameters are inner diameters.

In our experiments, we used a triple orifice spinneret for the spinning of hollow fiber membranes. The dimensions of the spinneret are shown in Figure 4. The dope solution was transported through the outer ring, the additive solution through the middle ring, and the bore fluid, a coagulant mixture of water and alcohol [25] (weight ratio 60/40), was transported through the inner orifice. The flow rates were varied and optimized accordingly to the membrane morphology, see Section 3.2.1. The spinning distance, i.e., the distance of the die to the coagulation bath with demineralized water, was set to 0.15 m. The polymer solutions were heated to 60 °C during spinning (see Table 2). The fibers were collected via turning the coagulation bath. After 30 min in the precipitation bath, the membranes were cut into 0.60 m long pieces. For complete precipitation, the membranes were rinsed with fresh demineralized water for at least overnight.

To wash out the PVP of the membrane matrix for well-interconnected pores and higher permeance, the hollow fiber membranes were post-treated with a solution of sodium hypochlorite of 2000 ppm, pH 9.5, adjusted via 1 M HCl, at a temperature of 60 °C for two hours. To remove active chlorine, the membranes were treated with a 0.5% solution of sodium bisulfite and washed withdemineralized hot water several times. All membranes were stored in water for further characterization.

### 2.6. Degree of Hydrophilicity

The hydrophilicity of the functionalized membranes was determined by contact angle measurements as well as indirectly by X-ray photoelectron spectroscopy (XPS, DLD Axis Ultra, Kratos, Manchester, UK) investigations of the chemical composition (O:S ratio) of the inner surfaces in comparison to the outer surface. XPS was carried out by an Al-K_α_ X-ray source (monochromator) operated at 225 W. For the survey spectra, a pass energy (PE) of 160 eV was used, while for the region scans, PE was 40 eV. Spectra were calibrated to the binding energy of the C1s of 284.5 eV, charge neutralization was necessary for all samples. Data analysis was carried out by CASAXPS V.2.3.18 software. For deconvolution of the region files, background subtraction (U2 Tougaard) was performed before calculation.

Contact angle measurements (Krüss Drop Shape Analyzer DSA100, Krüss GmbH, Hamburg, Germany) were carried out at room temperature using lengthwise cut hollow fiber membranes which were fixed on a glass plate. The sessile drop method was applied. To avoid the effect of surface porosity as much as possible, a high-speed analysis mode with 45 frames per second was used for the investigations. The contact angle of each membrane was determined after 0.2 s and averaged over five measurements on different areas on the inner and outer surface.

### 2.7. Membrane Morphology and Performance

The morphology of the membranes was investigated via a scanning electron microscope equipped with a field emission source (FE SEM, MERLIN™, Carl ZEISS, Oberkochen, Germany). For the cross sections the hollow fiber membranes were fractured under cryogenic conditions and attached onto a sample holder, as described in Ref. [26]. Prior to observation the samples were sputtered with a thickness of 2 nm platinum. The acceleration voltage varied between 3 kV and 5 kV.

The performance of the hollow fiber membranes was determined in ultrafiltration experiments. The investigations were carried out in dead-end mode, since in most industrial applications the modules are used in dead-end-mode. For retention tests aqueous PEG-solutions (0.001%, 100 kDa, dispersity index 1.05) were chosen. Gel permeation chromatography (GPC) analysis was carried out on samples taken after 20 min of filtration time.

## 3. Results

### 3.1. Characterisation of Solutions

#### 3.1.1. Rheology

**Figure 5:**
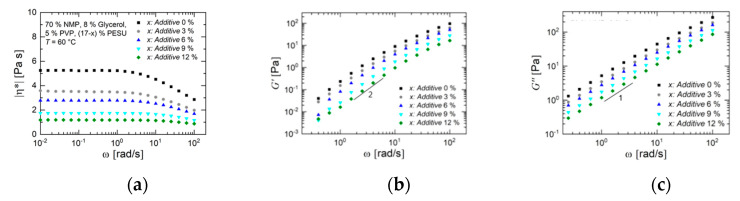
(**a**) Magnitude of complex viscosity as a function of angular frequency *ω* for solutions with additive concentrations of 0–12%. (**b**) Storage modulus *G’* as a function of angular frequency ω for these solutions. The data points at frequencies smaller than 0.3 rad/s are below the measurement resolution and thus are not shown. (**c**) Loss modulus *G″* as a function of angular frequency. The temperature was 60 °C.

**Figure 6:**
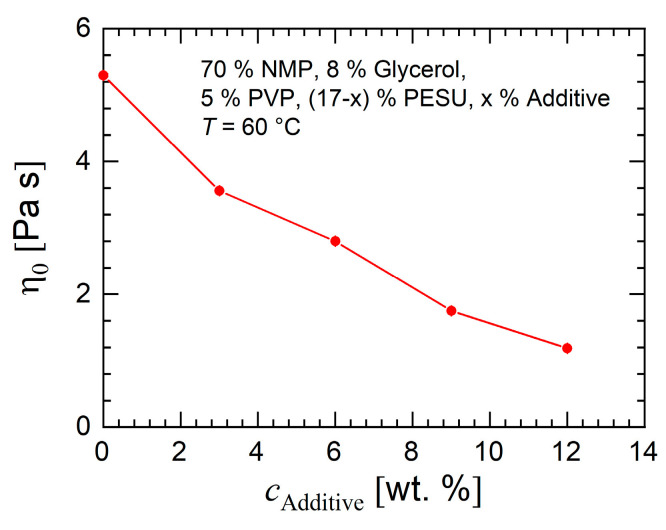
Zero shear rate viscosity $\eta_{0}$ as a function of additive concentration in solution at a temperature of 60 °C.

Investigations of the viscoelastic properties of the solutions reveal the influence of an increasing additive amount in the solutions. The magnitude of the complex viscosity (Figure 5a) in the frequency range between 0.01 to 100 rad/s decreased with increasing additive amount, which was caused by the low molecular weight of the additive. This trend is also seen for both dynamic moduli (Figure 5b,c) The solution AS 0 with no additive showed an approximately ten times higher value of storage modulus compared to AS 12 with 12% additive. The moduli seemed to follow a linear decrease with increasing additive concentration on the double-logarithmic presentation, which gives an idea of gradual lower relaxation times for shorter chains in the semi-dilute solution, caused by the low molecular weight of the additives. The data qualitatively agree with the Zimm model. The terminal regime of both dynamic moduli is clearly visible with characteristic slopes of 2 and 1, respectively. This allows the zero shear rate viscosity $\eta_{0}$ to be calculated which is displayed in Figure 6 and reveals a roughly linear trend with higher percentage of additives.

**Figure 7:**
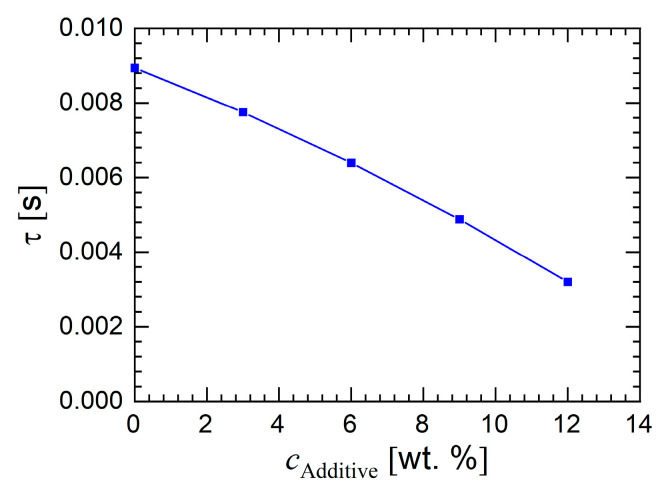
Average relaxation time of the additive solutions as a function of additive concentration.

The average relaxation time τ was determined by fitting the Cross model

(2) $$\left|\eta^{*}\left(\omega \right)\right|=\frac{\eta_{0}}{1+{\left(\tau \cdot \omega \right)}^{m}}$$

to the magnitude of complex viscosity. The average relaxation time *τ* is shown in Figure 7 and proposes a linear trend to faster relaxation of the chains in solution with increasing additive content. The shorter and less entangled the molecular chains, the shorter is the relaxation time. This result appears in shear deformation where a certain mechanical stress acts on the solution and where concentration fluctuations are negligible. Furthermore, possible agglomerated polymer chains may be broken up in shear flow.

#### 3.1.2. Dynamic Light Scattering

Dynamic light scattering is a method, which is sensitive to molecular agglomeration, formation and interaction in the absence of mechanical stress. In DLS experiments, the normalized field correlation function $g_{1}\left(q,t\right)$ can be determined when measuring the time-dependent intensity autocorrelation function $g_{2}\left(q,t\right)$ at a certain scattering vector q and reads, applying the Siegert relation, as follows:

(3) $$g_{2}\left(q,t\right)=1+\beta \;{\left|g_{1}\left(q,t\right)\right|}^{2}$$

with β being the instrument specific coherence factor, ranging between 0 and 1. Since the normalized field correlation function $g_{1}\left(q,t\right)$ is described by a spectrum of relaxation times H(τ) and relaxes with time t [27], it can be written as:

(4) $$g_{1}\left(q,t\right)=\int_{0}^{\infty }H\left(\tau \right)\exp\left(-t/\tau \right)d\ln\tau$$

**Figure 8:**
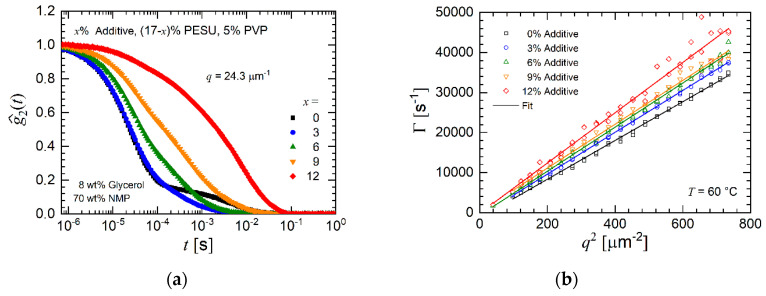
(**a**) Normalized intensity autocorrelation as a function of time for the additive solutions AS 0 to AS 12 at a constant value of the scattering vector. The measurement temperature was 60 °C. (**b**) Relaxation rate for each additive concentration as a function of the square of the magnitude *q* of the scattering vector for the fast relaxation process.

Rewriting Equation (3) yields the normalized intensity autocorrelation function as denoted by ${\hat{g}}_{2}\left(q,t\right)=\left[g_{2}\left(q,t\right)-1\right]/\beta$. In Figure 8a this function ${\hat{g}}_{2}$ is shown for a constant scattering vector of 24.3 µm^−1^. The solution AS 0 does not contain any additive and is associated with two clearly pronounced relaxation processes. In a previous work [23] it was shown that the second, slower process only appears in the presence of glycerol. An increasing additive concentration (solutions AS 3 to AS 9) leads to a slower decay of the function ${\hat{g}}_{2}$. The solution AS 12 is associated with an even slower decay. This solution also phase separates. The decay at short times is a clear diffusion process as seen by the pronounced linear trend of the relaxation rate as a function of *q*^2^.

**Figure 9:**
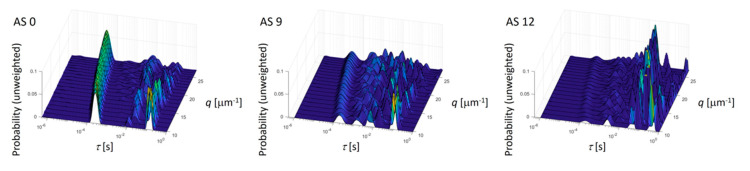
Relaxation time spectrum of the solutions AS 0, AS 9, and AS 12 as determined by the CONTIN algorithm for all measured scattering vectors.

The diffusion coefficient of the fast relaxation process, caused by collective diffusion, only moderately increases with additive concentration, see Figure 8b and Table 3. The reason for this trend is the low molecular weight of the additive, which yields a higher mobility than the pristine PESU chains. Figure 9 depicts the relaxation time spectrum for various scattering vectors and clearly shows that with increasing additive concentration the intensity of the very short relaxation process diminishes and peaks at longer relaxation times appear. The very fast relaxation process appears with almost no experimental scatter, whereas the slower relaxation processes scatter to a much larger extent. Consequently, longer relaxation times, according to the DLS measurements, become more dominant with increasing additive content in the solution. In contrast to the very monotonic variation of the peak at short relaxation times, the peaks at longer relaxation times appear more irregular. These peaks cannot be associated with a diffusion process because of their non-uniform appearance. Hence, no diffusion coefficient was determined for the slow processes.

This result can imply a local phase separation of the additive and the other polymers in the solution. Local phase separation of the solutions AS 3 to AS 9 appears because of concentration fluctuations. These concentration fluctuations cause local, temporary phase separation and are more likely with increasing additive concentration. The authors suggest an agglomeration similar to micellization where the additive surrounds larger molecules of a specific kind and temporarily builds domains within the solution. These domains are below visible level. In the rheological experiments at higher frequencies, these fluctuating agglomerates are destroyed and thus are not detected by the measurement of the viscosity. Agglomerates behave like large molecules with a low diffusion coefficient and therefore result in slow relaxation times.

#### 3.1.3. Cloud Point Determination

**Figure 10:**
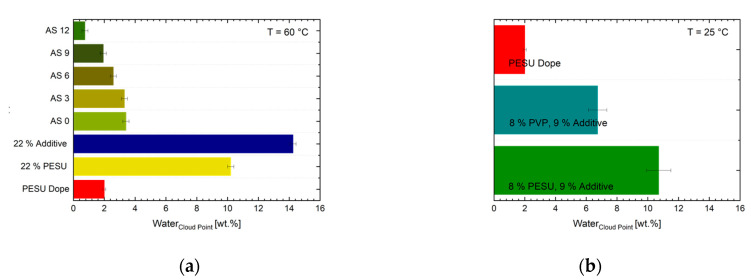
(**a**) Results of cloud point measurements for AS 0-12 and pure additive and PESU in NMP (both 22%) as well as the PESU standard solution, measured at 60 °C. In (**b**) the CP for PESU (8%) with additive (9%) and PVP (8%) with additive in NMP, as well as the PESU dope solution at 25 °C are compared. All CP display the mean value of three measurements for each sample.

**Figure 11:**
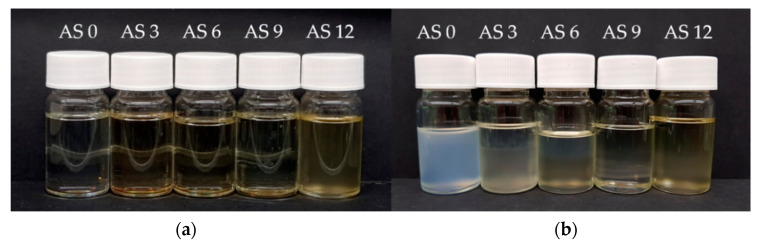
(**a**) Photograph of additive solutions AS 0 to AS 12 at room temperature. (**b**) All solutions after CP measurements. The solutions were not stirred for 24 h until the polymer rich phase (lower) macroscopically separated from the phase with low molecular weight species.

Figure 10 presents the cloud points of the solutions. By focusing on the cloud point (CP) of pure additive and pure PESU in NMP, one could assume that the addition of the additive of this study to a solution (while reducing the amount of PESU likewise) would result in higher cloud point values. Pure additive of 22% in NMP tolerates 14.3% water before turning turbid, while 22% of PESU in NMP becomes cloudy with roughly 4.0% less water resulting in a cloud point of 10.7%. Since we reduce, the amount of “fast” precipitating PESU while surrogating with additive, one could expect an even more pronounced effect to higher cloud points with increase of additive content. In fact, the results for AS 0 to AS 12 prove otherwise. With increasing additive amount and likewise reduced PESU the cloud point appears at lower concentrations. Furthermore, at room temperature AS 12 is turbid and phase separated right away, see Figure 11a. At the spinning temperature of 60 °C, the phase separation of AS 12 occurred only when adding 0.8% of water at 60 °C.

**Figure 12:**
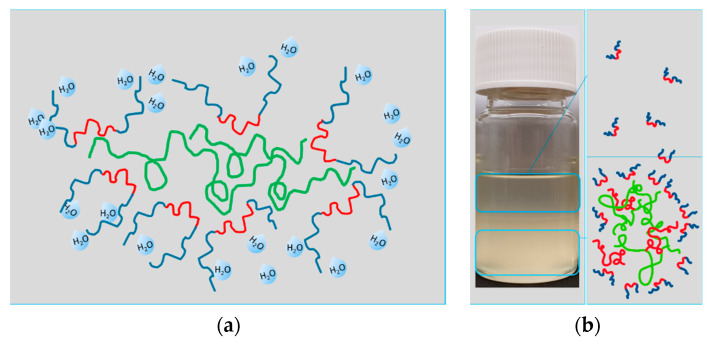
(**a**) Suggested agglomeration of PVP (green) in the inner and amphiphilic additives (red and blue) on the outer sphere. Here, a proposed state after cloud point measurements is illustrated. In (**b**) the macroscopic observed phase separation is sketched. Large molecules in red depict chains of PESU. All polymers are dissolved in a mixture of NMP, glycerol and water.

The data reveal an effect that is already suggested by the DLS results. The authors propose a mechanism that is known in solutions containing non-ionic surfactants and PVP or PEG molecules of high molecular weights [28]. We expect the PEO-blocks of the additives to be repelled by the PVP molecules of higher molecular weight, due to depletion flocculation. The depletion flocculation is well known in food science, especially in emulsions [29,30,31,32]. The assembly that is suggested works as a shielding of PVP molecules by block copolymers. The result is a locally phase separated solution, containing one high molecular weight (polymers like PVP and PESU) rich phase and above a less viscous phase with a higher fraction of species of low molecular weights. In our case, it is conceivable that the hydrophilic PEO parts surround those agglomerates and are pronounced in the phase consisting of NMP, glycerol, and water as well as detached additive molecules, as sketched in Figure 12. As a result of this mechanism at 60 °C, and preordering within the solutions with increased additive content, the visible phase separation that is induced by impairing the solvent through the addition of water, occurs a lot easier whenever larger or more agglomerates exist in the first place. The formation of PVP in the inner part and not PESU being encapsulated was proven by the cloud point investigations as depicted in Figure 10b, where PVP (8%) with additive (9%) as well as PESU (8%) with additive (9%) are tested for comparison. The temperature of 25 °C was chosen in order to increase the experimental accuracy by reducing water evaporation in the container at higher temperatures. However, pure PVP does not precipitate, but dissolves in water, in combination with additive the solution reveals a cloud point at 6.7% of water, while the same combination with PESU instead of PVP and additive, phase separates at a water content of more than 10.0%. In comparison to PESU, the presence of PVP depresses the CP.

#### 3.1.4. Precipitation Experiments

**Figure 13:**
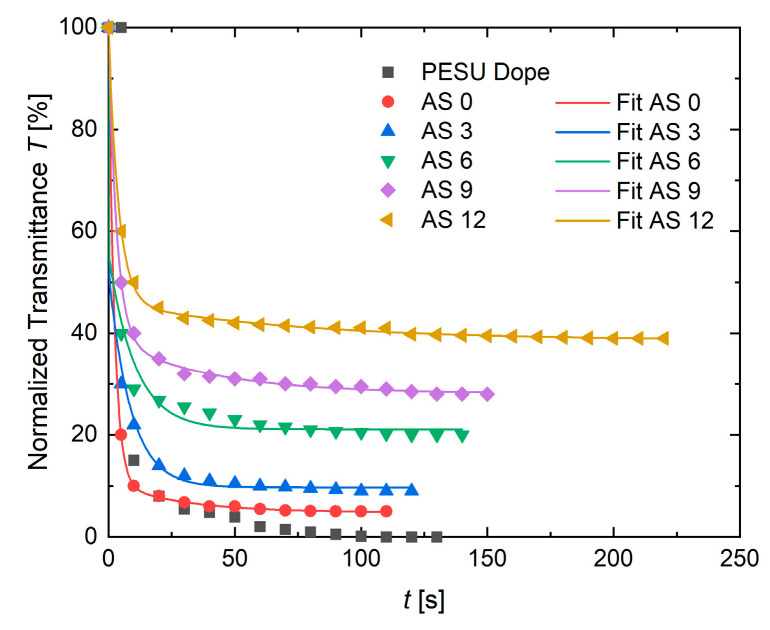
Transmission profiles for additive solutions containing 0–12% additive and for the PESU Dope solution. All measurements were carried out at room temperature.

The results of precipitation measurements are shown in Figure 13. It is obvious that the observed transmittance intensity increases with increasing additive concentration. This result can be explained by the decreasing thickness of the membranes, since the thicker the membrane, the more the light is absorbed. The evaluation was carried out with respect to a constant transmission value for at least 30 s. Only if one value did not change for at least 30 s, was the coagulation considered to be completed. The authors are aware that the final precipitation takes a lot more time than measured here. After the initial partial phase separation, the membrane is quite dense, already. The exchange of solvent and non-solvent mainly proceeds via diffusion. Nevertheless, the casted flat sheet membranes detach from the glass plate after a certain time. When this happens, the transmission measurement must be quitted on that membrane and repeated. Detached membranes deform in water and the measurement distance is changed by the detachment, obviously. The transmittance is very sensitive to the detector–sample distance, therefore this distance must be the same for all samples. The precipitation speed seems to be quite similar for all additive solutions in the first minute. It is in the second time interval that AS 12 differs a lot from all other solutions. Probably the first instantaneous demixing, in which the main porous structure is formed, occurs during the first 20 s in all solutions. Afterwards a delayed, slower solvent exchange out of the pores can take place and results in even more precipitated structures that are less transparent. Pores filled with a mixture of solvent and non-solvents (NMP/glycerol/water) are very likely. If the concentration of NMP is too high to precipitate more polymer chains, then the precipitation only proceeds either very slowly or due to diffusion processes. For additives that are no longer involved in the encapsulation of PVP molecules, a precipitation process starts that is depicted by the cloud point result for pure additive: here, a very high amount of water is needed to solidify the additives from solution. The second process explains why with increasing additive concentration the observed precipitation time increases. The results are supported by the phase separation that occurs during the cloud point measurements. One can see the polymer-rich phase (matrix structure forming species) being turbid and phase separated, while a transparent, yellowish phase (residual additive in solvent and non-solvents) is not yet precipitated. This explains the two ongoing processes in the solutions. The solutions AS 0 to AS 9 behave quite similarly. The remarkably extended precipitation of AS 12 is explained likewise by the high amount of additive that is solved in the upper transparent phase in the cloud point measurement and needs more water/time (CP/transmittance) to phase separate. Assuming a two phase precipitation process, the chosen fit function is based on the sum of two exponential decay functions:

(5) f(t)=A e−tτ1+B e−tτ2+C

with amplitudes A, B, and C as the offset and $\tau_{1,2}$ describing the two time constants. The fit parameters are shown in Table 4. With $\tau_{1,2}$ being the precipitation time constants, the trend to longer precipitation times as soon as additive is added to the composition (AS 3 to 12) is clearly represented in the data. AS 0 does not contain any additive and therefore other precipitation dynamics were measured. The multicomponent agglomerated domains that were described in the previous Section 3.1.3 are not formed when no additive is involved in the solutions. Hence the precipitation dynamics differ from those containing additives.

### 3.2. Characterisation of Membranes

#### 3.2.1. Membrane Morphology

The components of the solutions (see Table 1) affect the properties and the structure formation process as follows: PESU favors precipitation at a low water content, PVP in pure form is soluble in water and raises the viscosity as well as glycerol which acts as non-solvent and therefore decreases the solubility of the polymers in solution [23,33,34]. The PESU-PEO additive decreases the viscosity of the solutions due to its lower molecular weight, decreases the precipitation speed due to its amphiphilic nature and the solubility of the PEO, and of course makes the membrane hydrophilic.

**Figure 14:**
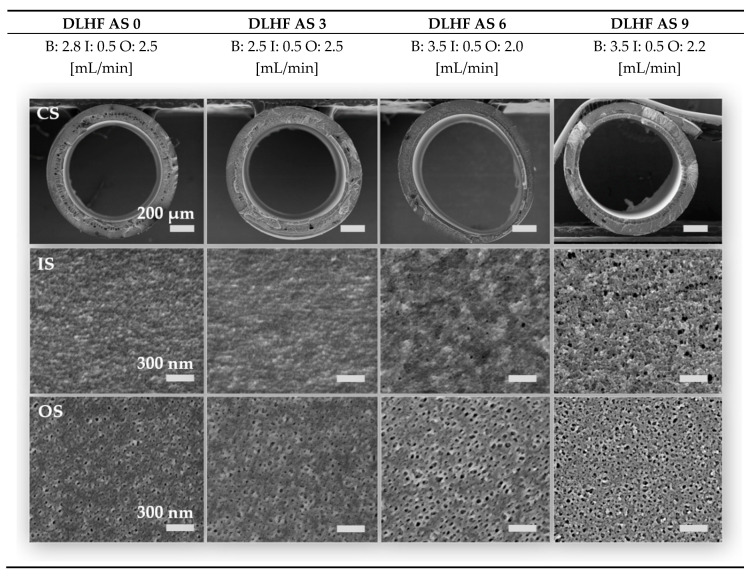
Micrographs of dual layer hollow fiber membrane DLHF AS 0 to AS 9. Flow rates of bore (B) fluid, inner additive solution (I) and outer polymer solution (O) refer to rates in mL/min. CS is the cross sectional view, IS abbreviates inner surface and OS outer surface. Due to agglomerated complexes in solution AS 12, AS 12 is only available as a flat sheet in order to prevent the spinneret from blocking.

**Figure 15:**
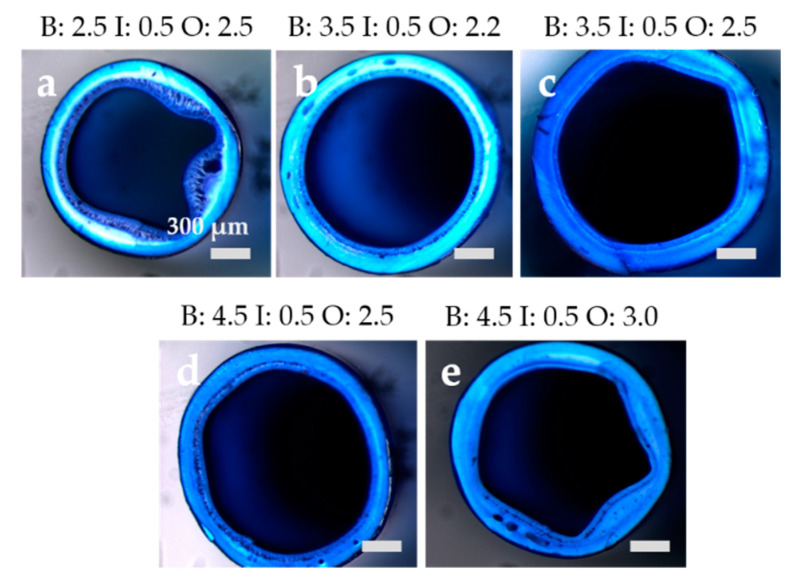
A selection of spinning parameters (bore (B) and outer (O) flow rates varied, while keeping the inner (I) flow rate) and resulting morphologies of DLHF AS 9. For better visibility of the porous structure in light microscopy, the fibers were dipped into 1% methylene blue in ethanol.

The PESU standard solution was adjusted in a way that was previously described by Cabasso et al. [35]. A high viscosity is desired to prevent the formation of macrovoids and results in a spongy, but still permeable and interconnected porous structure. By adding a high molecular weight additive like the PVP in this study as well as a non-solvent to worsen the solubility of PESU in the solvent mixture, one can reach the same viscosity increase as by increasing the PESU concentration. Another option, using either higher PESU concentrations (see also Ref. [33]) or PESU of higher molecular weight, would lead to defect-free, but rather dense membranes. Despite the fact, that the PESU standard solution is more viscous than all additive solutions, the previous analysis showed that the precipitation process of additive solutions AS 0 to AS 9 occurs on a similar timescale as the dope solution. This is useful for the production of dual layer membranes, since delamination of two layers often is more pronounced due to different precipitation times of those layers. Not only is the precipitation time of relevance, but also the spinning parameters can help to force the two layers onto each other during the precipitation process. In Figure 14 scanning electron micrographs of the cross sections (CS), inner and outer surfaces (IS and OS) of DLHF membranes AS 0 to AS 9 are displayed. The flow rates in mL/min of bore (B), inner slit with additive solution (I), and outer slit transporting the PESU dope solution (O) are shown as well. AS 12 was not spun due to its phase separated state in order to prevent the inner slit of the spinneret from blocking through agglomerates. Instead, flat sheet membranes were produced to investigate all additive solutions. These flat sheet membranes only consist of additive solution and do not have two layers. However, the flat sheet membranes allow a comparison of the porous structure, defects, and the hydrophilic character of the membranes. The higher the content of PESU, the more homogeneous and spongier is the pore structure of the inner additive layer. Due to the low molecular weight of the additive, a high additive content leads more likely to macrovoids. Furthermore, there was only a very tiny gap in spinning parameters, where no delaminated and round fibers could be achieved, see Figure 15. Within the flow rate ratios a phenomenological trend was observed, where the total of the polymer flow rate being higher than the bore flow rate (see, e.g., Figure 15a) led to a delaminated structure and collapse of the inner layer. This effect is probably observed, if the flow rate of the bore fluid is too low within the fiber and stabilizing precipitation does not occur fast enough on the outer layer. Instead, the inner layer solidifies while the supportive outer layer is still liquid, the solid inner layer than detaches from the outer part as soon as the fiber dives into the coagulation bath and dragged-like bridges can be observed between the two layers.

This detachment is prevented by choosing a high bore fluid flow rate (roughly 1 mL/min more than the sum of polymer flow rates) and assuring that the inner pressure and coagulation liquid exchange is high enough such that not only the inner layer solidifies within the spinning gap, but also the first part of the outer layer. Simultaneous precipitation is preferably aspired. Collapsed structures could also be avoided by adjustment of the bore fluid flow rate. The higher the bore fluid rate, the more likely circular fibers can be produced [36]. A general rule of thumb for prevention of starlike collapse in spinning of DLHF could not be observed. Nevertheless, it became obvious that the fiber spinning process and the adjustment of the flow rates is less sensitive to the bore:outer dope flow ratio with decreasing additive content. Probably, this happens to be the case because the precipitation with lower additive concentration is faster and the additive layer stabilizes quickly. This prevents the layer from delamination. Hence, a lower bore flow rate for low-concentrated additive solutions (AS 0 and AS 3) was chosen compared to the flow rate for the higher additive contents (AS 6 and AS 9). This is obvious since the additive layer and the outer layer become more similar in their composition with decreasing additive concentration and therefore achieve a higher viscosity while precipitation times remain alike. The technically lowest possible flow rate was 0.5 mL/min, which is the reason for the inner flow rate being set to 0.5 mL/min, since the additive layer should be as thin as possible.

**Figure 16:**
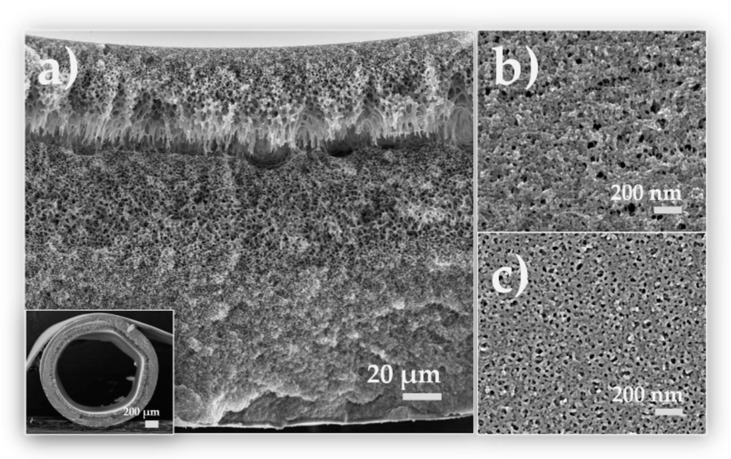
Exemplary porous structure of DLHF AS 9. (**a**) shows a magnification (356×) of the cross section (complete cross section with magnification of 40× in the inset) and the transient region between the support and the additive layer. At magnifications of 50,000× (**b**) depicts the inner pore distribution, while (**c**) features the outer porous structure.

The additive layer therefore resulted in a thickness of 50 µm for all membranes, the total wall thickness varied between 160 and 200 µm, depending on the outer dope flow rate. A higher bore fluid rate stretched the fiber from the inside and therefore resulted in a larger inner diameter in the range from 1100 µm at 2.5 mL/min to 1500 µm at a bore fluid rate of 4.5 mL/min (cf. Figure 15a–e)). A closer look into the pore structure of AS 9 in Figure 16 reveals a spongy asymmetric pore size distribution not only in the support layer, but also in the additive layer. The transient region between inner and outer layer offers many visible bridging connections, only broken in the very front, where the cryogenic preparation method destroyed some connections. The pores in the separation layer b) are mainly between 2 and 10 nm in size, on the outer side they are distributed quite uniformly between 20 and 40 nm. Many pores of the active inner surface are either collapsed during the drying process or below the experimental resolution. Some membranes (see Figure 14) did not depict pores on the inner layer. The pure water permeance (PWP) was measured to be over 1300 L/(m^2^ h bar), regardless of their observed pore structure via SEM. Consequently, the membranes with no detectable inner pores are not necessarily dense, as proved by our investigations.

#### 3.2.2. Membrane Hydrophilicity

**Figure 17:**
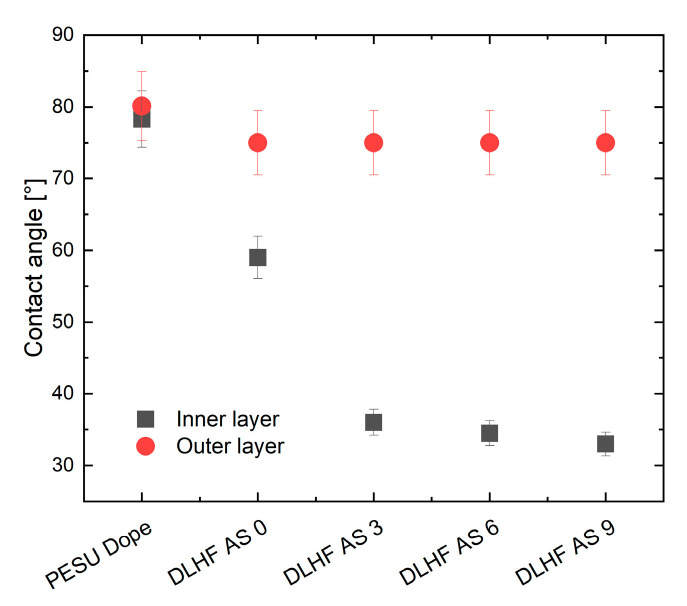
Results of contact angle measurements. Each value is the mean value of five contact angles on different areas of a sample. The hollow fiber membranes were tested on the inside and the outside. For AS 12 only the flat sheet membrane was available, which is why no contact angle value is presented here.

**Figure 18:**
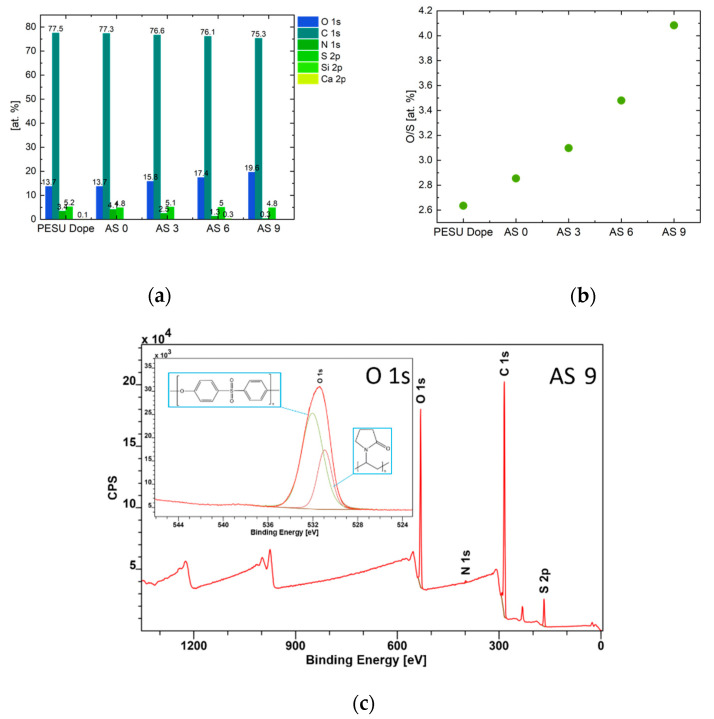
(**a**) Distribution of elements in at% (**b**) Ratio of oxygen to sulfur determined by XPS measurements. An exemplary low resolution XPS survey spectrum of AS 9 is shown in (**c**), while the inset displays the high resolution O 1s region.

As expected, the contact angle measurements as well as the investigations via XPS revealed an increased hydrophilic membrane character with higher additive concentration. The results are plotted in Figure 17 and Figure 18. It is a well-known challenge to interpret contact angles measured at membranes instead of films, knowing that the porosity affects the contact angle to a large extent. The authors therefore chose a sessile drop testing method in which the camera takes 45 pictures per second and evaluated the angle of the droplet in a constant region after 0.2–0.3 s. Thus, the porosity effect was eliminated as much as possible and weighted equally for all samples. In the exemplary XPS survey spectra of AS 9, see Figure 18c, C, O, and S were identified as main elements. The nitrogen peak depicts residues of PVP in the membrane structure after post-treatment. The high resolution spectrum of O 1s deconvolutes into two peaks at 530.95 eV and 532.04 eV binding energy. Sulfone groups are associated with the binding energy at approx. 532 eV as has been reported in [37], noting that differences of 0.5 eV may occur due to the calibration of the C 1s reference peak at 285.0 eV in their work and 284.5 eV in our investigations. Keeping in mind that the additive, as well as the PESU is built with sulfone groups, the large proportion of that peak seems reasonable. The peak at 530.95 eV corresponds to amide or nitrogen bonds with oxygen as well as to carboxylic acid, as proven by [38,39]. The authors therefore associate the peak at 530.95 eV with pyrrolidone of PVP and the oxyethylene. For analysis of the surface hydrophilicity the authors focused on the oxygen to sulfur ratio (O:S), obtained by the at% of these elements. With increasing amount of additive, the sulfone groups of PESU should be covered by the hydrophilic end-groups of the additives and therefore the O:S ratio has to increase. This effect was measured on all samples, as illustrated in Figure 18b.

#### 3.2.3. Membrane Performance

The results of the ultrafiltration experiments are shown in Table 5. In summary, the average PWP value of DLHF AS 9 was 2000 L/(m^2^ h bar) while the MWCO was measured to be 100 kDa. All membranes were post-treated as described previously in Section 2.5. before the ultrafiltration experiments, since otherwise the PVP content would prevent the pores from being interconnected and no or only a very low permeance would be observed. A higher content of low molecular weight species led to more open structures in the separation layer, resulting in higher permeances and higher MWCO. The high MWCO, as well as the increase in permeance measured for DLHF AS 6 connote defects in the membranes. Improved properties for this lot are likely possible after adjusting the spinning parameters. Comparable PWP for DLHF AS 9 and contemporary lower MWCO reveal a defect free structure for the membrane with the highest hydrophilicity.

## 4. Conclusions

Hydrophilic functionalized dual layer hollow fiber membranes were successfully produced via implementation of amphiphilic additives to the inner layer. Addition of PEO-PESU-PEO additives to the solutions was investigated at different concentrations of additive. A larger amount (12% in solution) of additive led to phase separation of the solutions due to depletion flocculation. Thus, membranes fabricated with a high additive content (9%) but still homogeneous solution were chosen for ultrafiltration experiments and revealed a PWP value of 2000 L/(m^2^ h bar) and a MWCO of 100 kDa. Consequently, the new hydrophilized dual layer hollow fiber membranes are promising candidates and not only for further fouling tests. An exchange through another additive while keeping all parameters constant is likely possible and an easy way to tune the active surface of the membranes onto desired charges conceivable. This makes the presented membranes interesting for various industrial purposes such as dams or sewage treatment plants.

## Figures and Tables

**Table 1:** Recipes of additive solutions as well as the outer dope solution. PESU denotes poly(ether sulfone). All values are given in wt%.

Solution	Additive	PESU	PVP	Glycerol	NMP
AS 0	0	17	5	8	70
AS 3	3	14	5	8	70
AS 6	6	11	5	8	70
AS 9	9	8	5	8	70
AS 12	12	5	5	8	70
PESU Dope (outer)	0	19	6	10	65

**Table 2:** Spinning parameters used in this study.

Parameter		DLHF AS 0	DLHF AS 3	DLHF AS 6	DLHF AS 9
Bore fluid	[mL/min]	2.8	2.5	3.5	3.5
Outer dope	[mL/min]	2.5	2.0	2.0	2.2
Additive solution	[mL/min]	0.5	0.5	0.5	0.5
Spinning temperature	[°C]	60			
Distance to coagulation bath	[cm]	15			

**Table 3:** Calculated diffusion coefficient *D* of the fast relaxation process of the polymer solutions AS 0 to AS 12 at 60 °C.

Solution	*D* [10^−12^ m^2^/s]
AS 0	48.4 ± 0.3
AS 3	52.3 ± 0.3
AS 6	55.1 ± 0.7
AS 9	54.7 ± 0.7
AS 12	62.8 ± 1.7

**Table 4:** Fit parameters for results of the transmission experiments.

	AS 0	AS 3	AS 6	AS 9	AS 12
A	89.20	47.01	43.39	60.79	53.85
τ_1_ [s]	2.30	0.09	0.06	3.16	3.88
B	6.00	42.32	34.47	11.06	7.48
τ_2_ [s]	27.85	8.69	10.47	38.25	73.08
C	4.80	9.72	21.09	28.12	38.61

**Table 5:** PWP and MWCO of DLHF membranes and the PESU standard dope membrane.

Membrane	PWP [L/(m^2^ h bar)]	MWCO [kDa]
DLHF AS 0	187	40
DLHF AS 3	578	60
DLHF AS 6	2040	200
DLHF AS 9	2000	100
PESU Dope	1200	50
